# Self-Perception and Assessment of Antibiotic Therapy Knowledge in Dental Students in Spain: A Cross-Sectional Observational Study

**DOI:** 10.3390/antibiotics14080755

**Published:** 2025-07-27

**Authors:** Ángel-Orión Salgado-Peralvo, Naresh Kewalramani, Irene-Alexandra Boullosa-Bernárdez, Carlos Oteo-Morilla, Ana-Leticia Lenguas-Silva, María-Rosario Garcillán-Izquierdo, María-Victoria Mateos-Moreno

**Affiliations:** 1 Department of Surgery and Medical-Surgical Specialties, Faculty of Dentistry and Medicine, University of Santiago de Compostela, 15782 Santiago de Compostela, Spain; 2 Department of Nursing and Stomatology, Faculty of Dentistry, Rey Juan Carlos University, 28008 Madrid, Spain; k93.naresh@gmail.com; 3 Department of Endodontics, Faculty of Dentistry, Mississippi University of Madrid, 28010 Madrid, Spain; irenealexandra@uic.es; 4 Department of Dental Clinical Specialties, Faculty of Dentistry, Complutense University of Madrid, 28040 Madrid, Spain; coteo@ucm.es (C.O.-M.); alenguas@ucm.es (A.-L.L.-S.); rgarcillan@ucm.es (M.-R.G.-I.)

**Keywords:** surveys and questionnaires, antimicrobial stewardship, anti-bacterial agents, tooth diseases, infection control, dental, students, dental

## Abstract

**Background**: The development of antimicrobial resistance is a major public health issue, in which dentists play a significant role by prescribing 7–11% of worldwide antibiotics. The aim of this study is to evaluate the self-perception and knowledge of antibiotic therapy in fifth-year undergraduate dental students. **Methods**: This is a cross-sectional observational study based on the STROBE (Strengthening the Reporting of Observational Studies in Epidemiology) guidelines. An electronic survey consisting of 18 questions was conducted with fifth-year students enrolled in the 2022/23 and 2023/24 academic years. The data were analyzed using descriptive and inferential statistical methods. **Results**: A total of 139 students (76.4%) completed the questionnaire. A total of 71.9% of students considered that they had received adequate education in antibiotic therapy, particularly in Oral Surgery (89.2%) and Periodontics (86.3%). The theoretical classes (3.50 ± 0.98) and practical sessions (3.18 ± 1.29) provided the knowledge that had the greatest influence on their education. They showed high self-confidence in diagnosing an infection (3.49 ± 0.73) and in choosing the appropriate antibiotic and dosage (3.26 ± 0.73). Over 76% of students answered correctly regarding the need for antibiotic prescriptions in various practical scenarios, except in the replantation of avulsed permanent teeth (54%). **Conclusions**: Dental students’ knowledge of antibiotics should be reinforced, as a high percentage answered correctly regarding the indications for antibiotics in pulpal and periapical diseases, but students performed less well regarding the choice of antibiotic and dosage in patients without sensitivity to β-lactams.

## 1. Introduction

Antibiotics in Dentistry can be used for (1) preventive purposes, to prevent systemic bacteremia in at-risk patients (antibiotic prophylaxis) or to achieve an adequate blood concentration to prevent bacterial contamination during surgical procedures (preventive antibiotic therapy) [1], or (2) for therapeutic purposes, in cases where an infection has already been established [2,3].

These drugs, along with local anesthetics and analgesic and/or anti-inflammatory drugs, are the most commonly used by dentists. However, they are often administered for prolonged periods, increasing the risk of adverse drug reactions, which are reported to exceed 1% following penicillin administration [4]. Nevertheless, the major issue arising from their use is the development of antimicrobial resistance (AMR). Specifically, AMR is responsible for over 700,000 deaths annually worldwide—approximately 25,000 deaths in the European Union (EU) and 35,000 in the United States (US)—and associated treatment costs and productivity losses amount to around 9 trillion EUR/year in the EU and 55 trillion USD/year in the US. It is estimated that the economic impact of AMR in 2050 will be equivalent to the 2008 financial crisis, at approximately USD 100 trillion globally [5]. By then, if immediate action is not taken, AMR will be responsible for 10 million deaths worldwide—the equivalent of the population of Sweden [6].

In this regard, global strategies for AMR prevention have been developed in recent years, such as the “One Health Concept,” promoted by international organizations like the World Health Organization (WHO), the Food and Agriculture Organization (FAO) of the United Nations, and the World Organisation for Animal Health [7]. In 2014, the Spanish Agency for Medicines and Health Products developed the Plan Nacional frente a la Resistencia a los Antibióticos (National Plan Against Antibiotic Resistance) [8] (PRAN), creating a working group in Dentistry in 2018. This milestone is essential because dentists are responsible for 7–11% of all global antibiotic prescriptions [9], of which 66% could be avoided [10], as many oral infections can be treated solely through local interventions that eliminate the source of infection [11]. Dentists must be familiar with the levels of AMR prevention. Primary prevention is crucial and involves not only the proper prescription of these drugs but also educating patients on their appropriate use and warning them about the risks associated with misuse [12]. Secondary prevention is based on the detection of AMR and its reporting to pharmacovigilance agencies, while tertiary prevention involves performing cultures and antibiograms in the presence of infections and identifying the appropriate antibiotics [3].

Within primary prevention, proper training of dental students is essential, as they will soon be authorized to prescribe antibiotics. In this regard, attitudes toward prescribing these drugs develop during undergraduate training and will influence students’ future clinical practice [11], as highlighted by a study where no differences were observed in prescribing patterns between junior and senior dentists [13]. In Spain, the bachelor’s degree in Dentistry curriculum (Bologna Plan) includes a mandatory Pharmacology subject in the third year as part of the Pathology and Medical Therapeutics module [14]. It is expected that by the end of their fifth year, students will be competent in prescribing medications, including antibiotics. Therefore, the aim of this study is to assess the self-perception and knowledge of antibiotic therapy acquired by fifth-year dental students at the Complutense University of Madrid (Madrid, Spain), to determine whether these skills need to be reinforced in the future.

## 2. Materials and Methods

### 2.1. Study Design

A cross-sectional observational study was conducted in accordance with the STROBE (Strengthening the Reporting of Observational Studies in Epidemiology) guidelines [15]. The study adhered to applicable legal and institutional regulations and received approval from the Ethics Committee of the San Carlos Clinical Hospital (Madrid, Spain) (Approval no. 25/273-E).

### 2.2. Hypothesis

The hypothesis of the study is that fifth-year undergraduate dental students will finish their Dentistry Degree with an appropriate knowledge of antibiotic therapy.

### 2.3. Questionnaire

A previously validated questionnaire that has been used in other studies [16,17,18,19,20] was employed, to which additional items were incorporated in order to assess students’ perceptions regarding the training received in antibiotic therapy throughout the Dentistry Degree at the Complutense University of Madrid (Madrid, Spain) (File S1). The questionnaire consisted of 18 mandatory items, designed such that respondents could not proceed to the next question without answering the current one. The Preventive and Community Dentistry Unit distributed the survey to fifth-year students by sending a Google Drive link to their institutional email addresses. Each student was permitted to complete the electronic survey only once, and submission of the questionnaire implied informed consent for data collection. All responses were anonymous, as no identifying information was requested. No financial incentives were offered for participation. Students were informed that they could withdraw their consent to participate in the survey at any time.

No selection bias was present, as the electronic survey was distributed to all students enrolled in the academic years 2022/23 and 2023/24.

### 2.4. Sample Size Calculation

The study population consisted of all fifth-year students enrolled during the academic years 2022/23 (n = 101) and 2023/24 (n = 81), totaling 182 students. Considering an analysis power of 90%, a confidence level of 95%, and a margin of error of 5%, a sample size of 125 respondents was calculated as ideal to detect statistically significant differences.

### 2.5. Statistical Analysis

The collected data were analyzed using IBM^®^ SPSS Statistics v.26 software (IBM^®^ Corp., Armonk, NY, USA). Descriptive and inferential statistics were utilized to report the general results of the study. For the descriptive analysis, frequencies and percentages were used, represented by pie charts; for ordinal qualitative data, frequencies and percentages, means, quartiles, medians, standard deviations, and minimum and maximum values were calculated and represented by bar charts. Contingency tables (with three categories of ordinal qualitative variables) were created, and either Fisher’s exact test (when expected frequencies were less than 5 in cells) or the Chi-square test was applied. To assess inference between variables, the non-parametric Kruskal–Wallis test was used for comparing more than two groups, followed by non-parametric pairwise comparisons with Bonferroni correction for *p*-values. Spearman’s correlation coefficient was employed to evaluate the relationship between pairs of variables. A *p*-value < 0.05 was considered statistically significant.

## 3. Results

### 3.1. Descriptive Data

The survey was completed by a total of 139 students (response rate of 76.4%), of whom 28 were men (20.2%) and 111 were women (79.9%).

### 3.2. Main Results Regarding Their Training in Antibiotic Therapy

**Figure 1:**
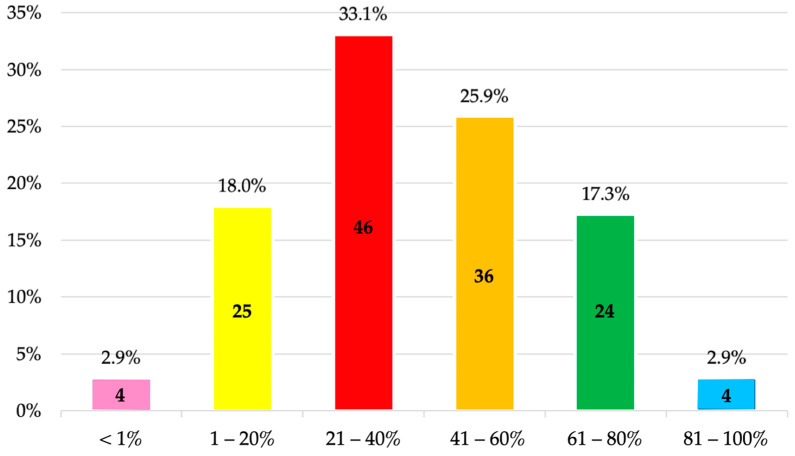
Percentage of prescriptions estimated by fifth-year undergraduate dental students to be unnecessary or potentially avoidable in Dentistry.

The majority of surveyed students (59%) consider that between 21% and 60% of antibiotic prescriptions are unnecessary or could have been avoided (Figure 1).

**Figure 2:**
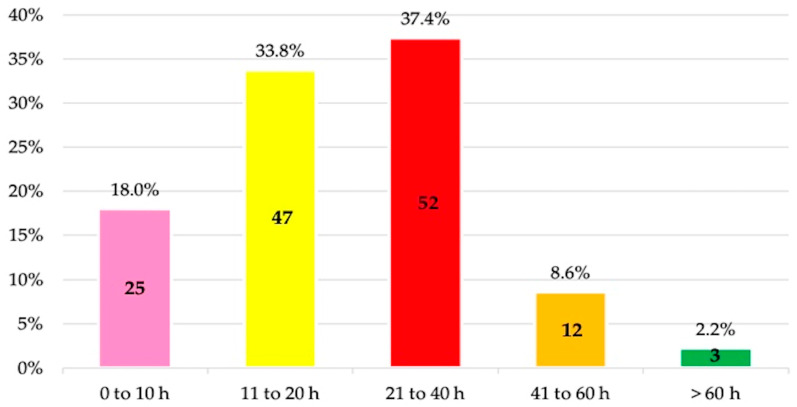
Estimated number of hours of training in antibiotic therapy during the undergraduate dental program by fifth-year undergraduate dental students.

A total of 71.2% reported having completed between 11 and 40 h of coursework on antibiotics throughout their Dentistry studies, specifically 33.8% between 11 and 20 h and 37.4% between 21 and 40 h (Figure 2).

The most utilized educational method was theoretical classes (98.6%), followed by clinical cases or practical scenarios (79.9%) and direct patient contact in the clinic (48.9%). Additionally, 23.0% of the students reported having used e-learning, and 23.7% considered it would have been a useful tool (Table 1).

In this regard, the knowledge that most influenced students was that acquired in theoretical classes (3.50 ± 0.98), followed by practical sessions (3.18 ± 1.29), and finally knowledge obtained outside the faculty (2.94 ± 1.16) (Table 2).

**Figure 3:**
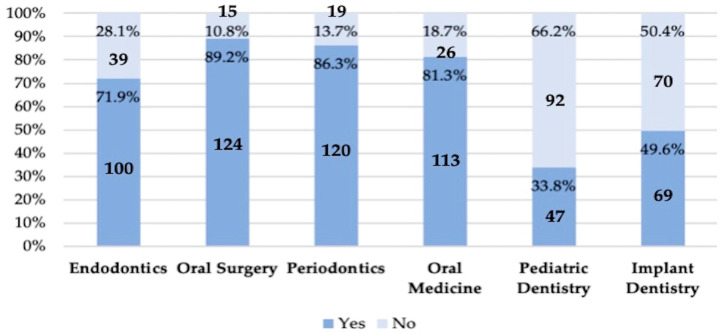
Fifth-year undergraduate dental students’ self-perceived knowledge across various thematic areas. Affirmative responses indicate adequate or sufficient knowledge.

Furthermore, 71.9% of students considered that they had been adequately trained in antibiotic therapy during their studies. The best-rated thematic areas were oral surgery (89.2%), periodontics (86.3%), oral medicine (81.3%), and endodontics (71.9%), while implant dentistry (49.6%) and pediatric dentistry (33.8%) were the lowest rated (Figure 3).

Overall, students reported feeling quite confident in diagnosing an infection upon completion of their Dentistry degree (3.49 ± 0.73) and in selecting the appropriate type and dosage of antibiotics (3.26 ± 0.73). There was a significant association with the influence of knowledge acquired outside the faculty (Pearson correlation [r] = 0.249; *p* = 0.003; and r = 0.200; *p* = 0.018, respectively), theoretical knowledge (r = 0.405; *p* < 0.001; and r = 0.270; *p* = 0.001, respectively), and clinical practice experience (r = 0.252; *p* = 0.003; and r = 0.208; *p* = 0.014, respectively). Additionally, students felt fairly confident managing a patient requesting antibiotic prescriptions without indication (3.55 ± 0.11).

A relevant finding was that only 40.3% of students were aware that antiseptics also contribute to bacterial resistance.

### 3.3. Knowledge About Antibiotic Therapy in Endodontics

Predominantly, the indications for which students tended to prescribe antibiotics were acute apical abscess with systemic symptoms (95.7%), progressing to cervicofacial cellulitis (95.7%), and asymptomatic cases in immunocompromised patients (82%), as well as reimplantation of avulsed permanent teeth (54.7%). Conversely, there was no consensus among students regarding antibiotic prescription for acute apical abscess without symptoms in healthy patients (18.7%), symptomatic irreversible pulpitis (pain) (8.6%), symptomatic apical periodontitis (19.4%), asymptomatic pulp necrosis (1.4%), and asymptomatic apical periodontitis (12.2%). The percentage of students who would prescribe antibiotics for various pulpal and periapical diagnoses is detailed in Table 3.

**Figure 4:**
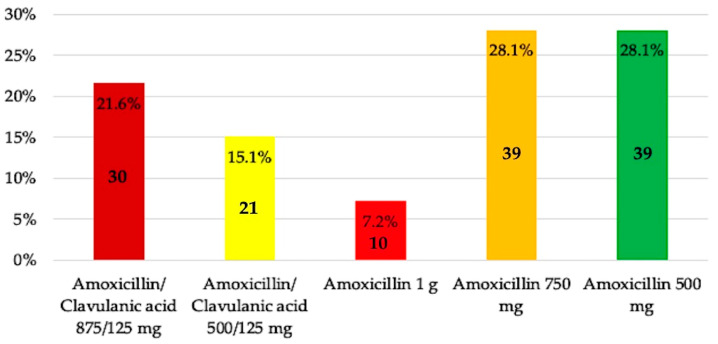
Antibiotic preference by fifth-year undergraduate dental students for patients without allergies.

When antibiotic therapy was indicated, the most commonly prescribed antibiotic in patients without allergies was amoxicillin 750 mg (28.1%) or 500 mg (28.1%), followed by amoxicillin/clavulanic acid 875/125 mg (21.6%) (Figure 4).

**Figure 5:**
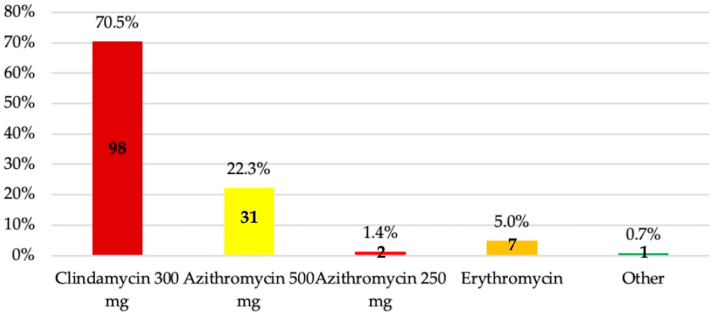
Antibiotic preference by fifth-year undergraduate dental students for patients with allergies.

In penicillin-allergic patients, clindamycin 300 mg (70.5%) and azithromycin 500 mg (22.3%) were the antibiotics of choice (Figure 5).

**Figure 6:**
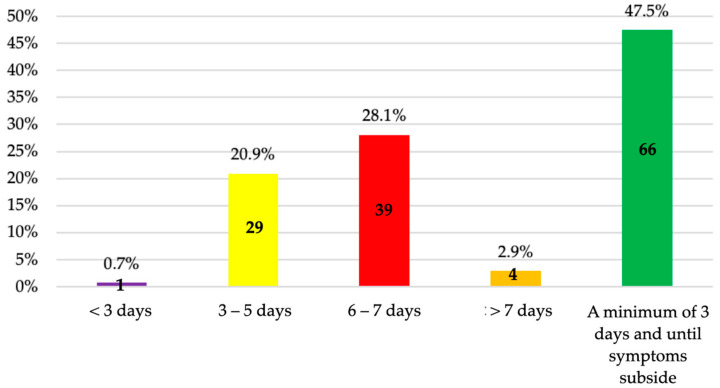
Duration of antibiotic treatment chosen by fifth-year undergraduate dental students.

The most frequently chosen treatment duration was a minimum of three days until symptoms resolved (47.5%), followed by 6–7 days (28.1%) (Figure 6).

Regarding this, 17.3% of students answered all three of these questions correctly, 44.6% answered two correctly, and 36% answered only one correctly. Only 2.2% answered all three incorrectly. No significant differences were found between the belief of having received adequate/sufficient knowledge on antibiotic therapy throughout their education and the correctness of responses concerning the type of antibiotic in patients with or without β-lactam allergies (*p* = 0.076 and *p* = 1.000, respectively), the treatment duration (*p* = 0.462), nor with the number of correct answers overall (*p* = 0.117).

## 4. Discussion

The findings presented in this study are of particular relevance as they serve two complementary purposes: first, to assess the perception that final-year undergraduate dental students have regarding the knowledge they have acquired; and second, to objectively evaluate that knowledge through specific questions focused on the indications and dosage regimens of antibiotic administration. The ultimate goal is to determine whether there is a need to reinforce antibiotic therapy content within the dental curriculum.

The survey was completed by a total of 139 students, of whom 28 were male (20.2%) and 111 female (79.9%). These figures are consistent with data published by the Spanish Ministry of Education [22], which reported that during the 2017/18 academic year, in the context of undergraduate education under the Bologna Framework, 52.3% of students enrolled in Spanish universities were women, compared to 33.8% men—thereby confirming the ongoing upward trend in female representation within Spanish higher education institutions.

Clinical scenarios related to pulpal and periapical pathology were proposed to evaluate this knowledge. The reason for using practical scenarios focused on endodontics was to narrow the scope of the study. In this regard, the European training program guidelines state that graduates should be adequately trained in the basic and clinical sciences of endodontics, enabling them to demonstrate competence in the understanding of the microbiology of endodontic-related pathologies and their management. In this context, students reported feeling confident when diagnosing a potential infection (3.49 ± 0.73), consistent with findings from students in the US [20] (mean confidence = 75.5%). This is reflected by the high percentage of students who correctly identified the indications for antibiotic prescription, which is in contrast with a study conducted in Peru that reported an insufficient level of antibiotic knowledge in 63.4% of students [23]. Therefore, there are still certain gaps that should be addressed through further training and incorporated into the dental curriculum.

According to the recommendations of the European Society of Endodontology [21] (2018), among the clinical scenarios presented, antibiotic administration would be indicated in (1) acute apical abscess with systemic involvement (localized fluctuant swellings, elevated body temperature >38 °C, malaise, lymphadenopathy, and trismus); (2) progressive infections (with rapid onset of severe infection, cellulitis or a spreading infection, osteomyelitis) where onward referral to oral surgeons may be necessary; and (3) acute apical abscess in medically compromised patients. These three indications were correctly identified by 95.7%, 95.7%, and 82% of the students, respectively, as the prescription of antibiotics is indicated in the presence of an infectious diagnosis [9,24]. In a study conducted in Egypt, 64.3% of respondents prescribed antibiotics for an acute facial swelling [25], compared to 86.6% in Saudi Arabia [13]. In a patient with a localized fluctuant periapical swelling without systemic involvement but with a condition compromising their immune system (such as leukemia, AIDS, advanced renal failure and/or dialysis, poorly controlled diabetes, patients undergoing radiotherapy or chemotherapy, patients receiving corticosteroid therapy, and patients on immunosuppressive therapy following transplantation), the level of neutropenia is the risk indicator and may be categorized as mild (1000 to 1500/μL), moderate (500 to 1000/μL), or severe (<500/μL) [26]. In the latter, antibiotic therapy is indicated [21], as the antibiotic supports the immune system in eliminating the bacteria [27].

In the case of an (4) acute apical abscess without symptoms in healthy patients, antibiotic prescription is not indicated [13,21,28]. In this scenario, the percentage of correct responses dropped to 77%, while in Egypt [25] and Saudi Arabia [13], 82% and 48.46% of students answered correctly, respectively. Likewise, (5) replantation of avulsed permanent teeth does warrant the administration of these drugs [21,29], as avulsed teeth may become contaminated. Therefore, the goal is to prevent infection and reduce the risk of inflammatory root resorption [30,31]. However, only 54.7% of students responded affirmatively.

On the other hand, in (6) irreversible pulpitis with moderate/severe symptoms or (7) symptomatic apical periodontitis, the pulp is still vital and patients do not exhibit systemic symptoms, so antibiotics are not indicated [16,28,32]. This was correctly answered by 88.5% and 76.3% of students, respectively. In other Spanish universities, these percentages drop to 71% and 37%, respectively [16], while in Morocco, 88–100% and 86–93% answered correctly, respectively [33]. In Egypt [25], Saudi Arabia [13], and Turkey [34], 13.4%, 27.69%, and 3.9% of students, respectively, reported prescribing antibiotics in cases of irreversible pulpitis.

Finally, a healthy patient with (8) asymptomatic necrotic pulp and/or (9) apical periodontitis does not require antibiotic treatment, as root canal therapy alone is sufficient [28,32]. This was correctly answered by 92.8% and 84.2% of students, respectively. In comparison, 84% of students from various Spanish universities [16], 89–99% and 42–84% in Morocco [33], and 95.1% in Turkey [34] answered correctly. Although the present study did not inquire about the need to prescribe antibiotics for postoperative pain, in Morocco, 7.5–22.8% of students reported prescribing them for this purpose [33], possibly as a response to patient pressure or diagnostic uncertainty [11,35,36]. This situation, however, can be adequately managed with oral corticosteroids [37]. A promising finding in the present study is that students reported feeling fairly confident when dealing with a patient requesting antibiotics inappropriately (mean score = 3.55 ± 0.11), which is in line with results from the US (mean confidence = 77%) [20]. In contrast, it is concerning that only 33.8% of students considered they had received adequate training in antibiotic therapy in Pediatric Dentistry, which may lead to inappropriate prescribing in children and the development of antimicrobial resistance from an early age.

Students reported feeling confident when selecting the appropriate type of antibiotic and dosage (3.26 ± 0.73), similar to findings in the US, where students expressed an average confidence level of 67.6% in choosing the type of antibiotic and 62.9% in selecting the dosage [20]. In this regard, acute or chronic endodontic infections are usually polymicrobial, with anaerobic species such as *Fusobacterium*, *Prevotella*, *Porphyromonas*, *Actinomyces*, *Streptococcus*, and *Peptostreptococcus* predominating, and typically located in the root canal system [38]. Therefore, when antibiotic prescription is indicated, current guidelines recommend administering a loading dose of 1 g of amoxicillin, followed by 500 mg every 8 h for the treatment of endodontic infections [21,28]. Based on these criteria, only 35.3% of students in the present study selected both the correct antibiotic and dosage. Currently, the rate of correct loading dose prescription ranges from 38% to 50% [34,39]. A similar multicenter study conducted among fifth-year dental students from four Spanish universities (Barcelona, Zaragoza, Santiago de Compostela, and Seville) showed that 44% of students chose amoxicillin as their first-line antibiotic. However, only 14.1% prescribed a dose of 500 mg and 3.5% a dose of 1 g, resulting in just 17.6% of students responding appropriately [16]. Amoxicillin is the most commonly prescribed antibiotic among dental students in Morocco (83.8%) [32], Saudi Arabia (88.4%) [13], and Turkey (86.7%) [34]. In Saudi Arabia and Turkey, the combination of amoxicillin with clavulanic acid is also widely used, which should be considered in cases of amoxicillin inefficacy due to β-lactamase-producing bacteria [13,34].

In patients allergic to β-lactams, it is important to confirm such sensitivity through specific diagnostic tests, since it has been shown that 10–20% of patients who claim to be allergic to penicillin rarely experience hypersensitivity or immunoglobulin E-mediated reactions and, therefore, these drugs could be safely used [40]. For this reason, between 80 and 99% of patients may no longer be considered allergic after undergoing β-lactam sensitivity testing [41]. If allergy is confirmed, several alternatives are available, such as clindamycin (a loading dose of 600 mg followed by 300 mg every 6 h), azithromycin (500 mg followed by 250 mg every 24 h), or clarithromycin (500 mg followed by 250 mg every 12 h) [21]. Accordingly, 94.2% of students who answered clindamycin 300 mg or azithromycin 500 mg or 250 mg responded correctly. In a Spanish multicenter study, 98.7% of students chose clindamycin 300 mg, while 0% and 1.3% selected azithromycin 250 mg and 500 mg, respectively [16]. In Morocco, the percentage of students who chose clindamycin was lower (53.9%), while 20.9% selected azithromycin [33]; in Turkey, 94.1% chose clindamycin as their first option [34].

Normally, endodontic infections have a rapid onset and short duration, resolving within 3–7 days or less if the cause is treated or eliminated [16]. As soon as the symptoms resolve and clinical improvement is observed, antibiotic therapy should be discontinued [21]. Most of the respondents (47.5%) would prescribe antibiotics for endodontic infections “a minimum of three days and until symptoms subside.” In the study conducted in Spanish universities, the same response options were not provided as in the present study. However, the average duration was 7.0 ± 2.0 days, with the most frequent regimen being 7 days (69%) [16]. In Morocco, the average duration was 5.87 ± 1.45 days, with a maximum of 7 days (56.5%) and a minimum of 3 days (14%) [33], while in Saudi Arabia, 69.3% prescribed antibiotics for 3–5 days [13], which aligns with the recommendations mentioned above.

The most frequently used educational methods were theoretical lectures (98.6%) and clinical case discussions (79.9%), while e-learning was used to a lesser extent (23%), which is in line with data from the US (97%, 55.5%, and 16.5%, respectively) [20]. Having a solid theoretical foundation is essential; however, direct patient interaction is considered the most helpful format for this topic of study [20,42]. Interactive small group exercises and patient education can also be very beneficial [43,44], allowing students to design educational materials for patients, engage in role-playing activities to practice real-life scenarios such as negotiating the need for antibiotics, and educate patients about proper antibiotic use. Preparing mini reviews on antibiotics or on their misuse by professionals and the development of AMR can also be useful [25].

In this regard, 59% of the surveyed students considered that between 21% and 60% of antibiotic prescriptions are unnecessary or could have been avoided, and 33.8% reported having received 11 to 20 h of training, while 37.4% had received between 21 and 40 h. In the US, these figures are 35%, 41%, and 22.5%, respectively [20]. A key question that arises is whether this number of training hours is sufficient during dental studies to acquire the necessary competencies for the appropriate prescription of antimicrobials and the prevention of AMR. Therefore, it is essential to monitor learning outcomes at the university level. In this respect, not only cross-sectional studies have been conducted [11,13,16,17,18,20,24,33,36,45], but also longitudinal ones, such as the study by Badran et al. [25] (2022), who used the Kirkpatrick model to evaluate dental students’ knowledge of antibiotics and AMR at two different points in their studies (in the second and fourth years). These authors observed that correct responses regarding antibiotic therapy increased from 68.3% to 80% after the educational sessions and that knowledge related to AMR improved from 78.9% to 83.5%, respectively. Such initiatives are fundamental, as prior to the intervention, some students considered the prescription of these drugs necessary for the treatment of gingivitis (45.7%) and for common colds and influenza (40.7%), among other conditions. Nevertheless, the findings reported in these studies (including the present one) ultimately reflect the knowledge and teaching skills of the educators, as well as the educational resources provided by the universities. Therefore, these results should serve as an incentive to continue investing in both teaching materials and the continuous professional development of faculty members.

### 4.1. Limitations

This study has several limitations. First, the veracity of the data provided by respondents could not be verified. Second, because the sample was limited to fifth-year students from a single institution, the findings may not be generalizable to other national or international dental schools. Third, in the absence of a validated survey instrument on this topic, comparison of our results with those of previous studies is challenging, particularly given the variations in prescribing practices across countries and/or continents. Finally, the length of the questionnaire—designed to capture multiple variables—may have led some participants to discontinue before completion, and the large number of variables complicated the data analysis.

### 4.2. Recommendations for Further Research

Future studies should employ a standardized design to facilitate interstudy comparison and incorporate scoring criteria to evaluate the appropriateness of students’ antibiotic selections. Such methodological rigor is essential to accurately assess antibiotic-prescribing knowledge in dental students, who constitute future antibiotic prescribers.

## 5. Conclusions

Within the limitations of this study, it can be concluded that antibiotic-related knowledge in undergraduate dental education should be reinforced. Although a high percentage of fifth-year students correctly identified the indications for antibiotic use in pulpal and periapical pathologies, they demonstrated less accuracy in selecting the appropriate antibiotic type and dosage for patients without β-lactam sensitivity. It is essential to periodically assess students’ knowledge on antibiotic use in order to help reduce the future prevalence of antimicrobial resistance, especially considering that these students are future prescribers. Moreover, investment from relevant institutions is essential to support the continued education of university faculty responsible for training future dentists in this critical area.

## Figures and Tables

**Table 1:** Educational methods used during antibiotic instruction and their perceived usefulness by fifth-year undergraduate dental students.

Educational Method	Yes		No		No, But I Believe It Would Be Useful	
	N	%	N	%	N	%
Lectures	137	98.6	1	0.7	1	0.7
Clinical cases/simulations	111	79.9	13	9.4	15	10.8
Direct patient interaction	68	48.9	27	19.4	44	31.7
E-learning	32	23.0	74	53.2	33	23.7

N, number of students; %, percentage.

**Table 2:** Questions related to antibiotic education (1 = not confident at all; 2 = slightly confident; 3 = moderately confident; 4 = very confident; 5 = completely confident).

Question	Mean Score ± SD
How confident do you feel in making an accurate diagnosis of infection?	3.49 ± 0.73
How confident do you feel in prescribing antibiotics? (selecting the appropriate antibiotic and dosage)	3.26 ± 0.73
How confident do you feel when dealing with a patient who requests an antibiotic prescription when it is not clinically indicated?	3.55 ± 0.11
To what extent have theoretical classes influenced your knowledge and training in antibiotics?	3.50 ± 0.98
To what extent has clinical training influenced your knowledge and training in antibiotics?	3.18 ± 1.29
To what extent has your interest in the topic outside the formal undergraduate dental curriculum (e.g., self-directed learning, attendance at related courses or webinars) influenced your knowledge and training in antibiotics?	2.94 ± 1.16
Do you believe that dentists play a key role in the prevention of antimicrobial resistance?	4.56 ± 0.66

SD, standard deviation.

**Table 3:** Clinical situations in which antibiotics would be prescribed (correct answers according to the recommendations of the European Society of Endodontology [21] are marked in bold).

Clinical Situations	Yes		No		Don’t Know	
	N	%	N	%	N	%
AAA with systemic symptoms (fever, malaise, and/or lymphadenopathy)	133	**95.7**	5	3.6	1	0.7
AAA progressing to cervicofacial cellulitis	133	**95.7**	5	3.6	1	0.7
AAA without symptoms in healthy patients (localized swelling only)	26	18.7	107	**77.0**	6	4.3
AAA without symptoms in immunocompromised patients (localized swelling only)	114	**82.0**	18	12.9	7	5.0
Symptomatic IP (pain without signs or symptoms of pulpal or periapical infection)	12	8.6	123	**88.5**	4	2.9
Asymptomatic pulpal necrosis (no percussion or mastication pain)	2	1.4	129	**92.8**	8	5.8
IP with symptomatic apical periodontitis (spontaneous acute pain, percussion and mastication pain; widening of the periodontal ligament is observed)	27	19.4	106	**76.3**	6	4.3
Asymptomatic apical periodontitis (apical radiolucency with or without fistula)	17	12.2	117	**84.2**	5	3.6
Reimplantation of avulsed permanent tooth	76	**54.7**	46	33.1	17	12.2

N, number of students; %, percentage; AAA, acute apical abscess; IP, irreversible pulpitis.

## Data Availability

The data that support the findings of this study are available from the corresponding author upon reasonable request.

## Supplementary Materials

The following supporting information can be downloaded at: https://www.mdpi.com/article/10.3390/antibiotics14080755/s1, File S1: Questionnaire.

## References

[1] Salgado-Peralvo A.O., Kewalramani N., Garcia-Sanchez A., Peña-Cardelles J.F. (2022). “Antibiotic Prophylaxis” and “Preventive Antibiotic Therapy”: Two Sides of the Same Coin. J. Stomatol. Oral Maxillofac. Surg..

[2] Oberoi S.S., Dhingra C., Sharma G., Sardana D. (2015). Antibiotics in Dental Practice: How Justified Are We. Int. Dent. J..

[3] Salgado-Peralvo A.O., Kewalramani N., Mateos-Moreno M.V. (2023). Antibiotic Resistance: The Silent Pandemia (in the COVID-19 Era). Int. Dent. J..

[4] Bhattacharya S. (2010). The Facts about Penicillin Allergy: A Review. J. Adv. Pharm. Technol. Res..

[5] Review on Antimicrobial Resistance (2014). Antimicrobial Resistance: Tackling a Crisis for the Health and Wealth of Nations. https://amr-review.org/sites/default/files/AMR%20Review%20Paper%20-%20Tackling%20a%20crisis%20for%20the%20health%20and%20wealth%20of%20nations_1.pdf.

[6] World Health Organization (2020). Record Number of Countries Contribute Data Revealing Disturbing Rates of Antimicrobial Resistance.

[7] World Health Organization, Food and Agriculture Organization of the United Nations, World Organisation for Animal Health (2008). Contributing to One World, One Health. A Strategic Framework for Reducing Risks of Infectious Diseases at the Animal-Human-Ecosystems Interface.

[8] Agencia Española de Medicamentos y Productos Sanitarios (2014). Plan Nacional Frente a La Resistencia a Los Antibióticos 2014–2018.

[9] Dar-Odeh N.S., Abu-Hammad O.A., Al-Omiri M.K., Khraisat A.S., Shehabi A.A. (2010). Antibiotic Prescribing Practices by Dentists: A Review. Ther. Clin. Risk Manag..

[10] Roberts R.M., Bartoces M., Thompson S.E., Hicks L.A. (2017). Antibiotic Prescribing by General Dentists in the United States, 2013. J. Am. Dent. Assoc..

[11] AboAlSamh A., Alhussain A., Alanazi N., Alahmari R., Shaheen N., Adlan A. (2018). Dental Students’ Knowledge and Attitudes towards Antibiotic Prescribing Guidelines in Riyadh, Saudi Arabia. Pharmacy.

[12] Sukumar S., Martin F., Hughes T., Adler C. (2020). Think before You Prescribe: How Dentistry Contributes to Antibiotic Resistance. Aust. Dent. J..

[13] Iqbal A. (2015). The Attitudes of Dentists towards the Prescription of Antibiotics during Endodontic Treatment in North of Saudi Arabia. J. Clin. Diagn. Res..

[14] Agencia Nacional de Evaluación de la Calidad y Acreditación (2004). Libro Blanco Del Título de Grado En Odontología.

[15] Von Elm E., Altman D.G., Egger M., Pocock S.J., Gøtzsche P.C., Vandenbroucke J.P. (2008). The Strengthening the Reporting of Observational Studies in Epidemiology (STROBE) Statement: Guidelines for Reporting Observational Studies. J. Clin. Epidemiol..

[16] Martín-Jiménez M., Martín-Biedma B., López-López J., Alonso-Ezpeleta O., Velasco-Ortega E., Jiménez-Sánchez M.C., Segura-Egea J.J. (2018). Dental Students’ Knowledge Regarding the Indications for Antibiotics in the Management of Endodontic Infections. Int. Endod. J..

[17] Segura-Egea J.J., Velasco-Ortega E., Torres-Lagares D., Velasco-Ponferrada M.C., Monsalve-Guil L., Llamas-Carreras J.M. (2010). Pattern of Antibiotic Prescription in the Management of Endodontic Infections amongst Spanish Oral Surgeons. Int. Endod. J..

[18] Rodriguez-Núñez A., Cisneros-Cabello R., Velasco-Ortega E., Llamas-Carreras J.M., Tórres-Lagares D., Segura-Egea J.J. (2009). Antibiotic Use by Members of the Spanish Endodontic Society. J. Endod..

[19] Yingling N.M., Byrne B.E., Hartwell G.R. (2002). Antibiotic Use by Members of the American Association of Endodontists in the Year 2000: Report of a National Survey. J. Endod..

[20] Holz M., Naavaal S., Stilianoudakis S., Carrico C., Byrne B.E., Myers G.L. (2021). Antibiotics and Antimicrobial Resistance: Evaluation of the Knowledge, Attitude, and Perception among Students and Faculty within US Dental Schools. J. Dent. Educ..

[21] Segura-Egea J.J., Gould K., Şen B.H., Jonasson P., Cotti E., Mazzoni A., Sunay H., Tjäderhane L., Dummer P.M.H. (2018). European Society of Endodontology Position Statement: The Use of Antibiotics in Endodontics. Int. Endod. J..

[22] Ministerio de Ciencia Innovación y Universidades (2020). Estadística de Estudiantes. Estadísticas Universitarias.

[23] Requejo-Bustamante A.P., Perona-Miguel de Priego G.A. (2023). Level of Knowledge in Undergraduate Dental Students about the Prescription of Analgesics, Anti-Inflammatories, and Antibiotics in Pediatric Dentistry. Rev. Cient. Odontol..

[24] Guzmán-Álvarez R., Medeiros M., Lagunes L.R., Campos-Sepúlveda A. (2012). Knowledge of Drug Prescription in Dentistry Students. Drug Healthc. Patient Saf..

[25] Badran A.S., Keraa K., Farghaly M.M. (2022). Applying the Kirkpatrick Model to Evaluate Dental Students’ Experience of Learning about Antibiotics Use and Resistance. Eur. J. Dent. Educ..

[26] Schwartzberg L.S. (2006). Neutropenia: Etiology and Pathogenesis. Clin. Cornerstone.

[27] Mohammadi Z. (2009). Systemic, Prophylactic and Local Applications of Antimicrobials in Endodontics: An Update Review. Int. Dent. J..

[28] Segura-Egea J.J., Gould K., Şen B.H., Jonasson P., Cotti E., Mazzoni A., Sunay H., Tjäderhane L., Dummer P.M.H. (2017). Antibiotics in Endodontics: A Review. Int. Endod. J..

[29] Fouad A.F., Abbott P.V., Tsilingaridis G., Cohenca N., Lauridsen E., Bourguignon C., O’Connell A., Flores M.T., Day P.F., Hicks L. (2020). International Association of Dental Traumatology Guidelines for the Management of Traumatic Dental Injuries: 2. Avulsion of Permanent Teeth. Dent. Traumatol..

[30] Sae-Lim V., Wang C.Y., Choi G.W., Trope M. (1998). The Effect of Systemic Tetracycline on Resorption of Dried Replanted Dogs’ Teeth. Dent. Traumatol..

[31] Hammarström L., Blomlöf L., Feiglin B., Andersson L., Lindskog S. (1986). Replantation of Teeth and Antibiotic Treatment. Dent. Traumatol..

[32] Agnihotry A., Thompson W., Fedorowicz Z., van Zuuren E.J., Sprakel J. (2019). Antibiotic Use for Irreversible Pulpitis. Cochrane Database Syst. Rev..

[33] Khalaf L.H., Kabbaj S., Toure B. (2024). Attitudes of Dental Students towards the Prescription of Antibiotics during Endodontic Treatment. Antibiotics.

[34] Arıcan B., Çiftçioğlu E., Işık V., Karagöz-Küçükay I. (2021). Evaluation of the Knowledge of Final-year Dental Students on the Use of Antibiotics in Endodontics in Turkey. Aust. Endod. J..

[35] Yashin A.N., Thakuria N., Narzary H., Satnami D., Paul N. (2018). A Questionnaire Based Survey on the Knowledge, Attitude and Practices about Antimicrobial Resistance and Usage among the MBBS Students and Doctors of a Tertiary Care Teaching Hospital in Silchar, Assam, India. Int. J. Basic Clin. Pharmacol..

[36] Yusef D., Babaa A.I., Bashaireh A.Z., Al-Bawayeh H.H., Al-Rijjal K., Nedal M., Kailani S. (2018). Knowledge, Practices & Attitude toward Antibiotics Use and Bacterial Resistance in Jordan: A Cross-Sectional Study. Infect. Dis. Health.

[37] Jose J., Teja K.V., Palanivelu A., Khandelwal A., Siddique R. (2022). Analgesic Efficacy of Corticosteroids and Nonsteroidal Anti-Inflammatory Drugs through Oral Route in the Reduction of Postendodontic Pain: A Systematic Review. J. Conserv. Dent..

[38] Kassolis J.D., Scheper M., Jham B., Reynolds M.A. (2010). Histopathologic Findings in Bone from Edentulous Alveolar Ridges: A Role in Osteonecrosis of the Jaws?. Bone.

[39] Bolfoni M.R., Pappen F.G., Pereira-Cenci T., Jacinto R.C. (2018). Antibiotic Prescription for Endodontic Infections: A Survey of Brazilian Endodontists. Int. Endod. J..

[40] Trubiano J.A., Adkinson N.F., Phillips E.J. (2017). Penicillin Allergy Is Not Necessarily Forever. JAMA.

[41] Trubiano J.A., Thursky K.A., Stewardson A.J., Urbancic K., Worth L.J., Jackson C., Stevenson W., Sutherland M., Slavin M.A., Grayson M.L. (2017). Impact of an Integrated Antibiotic Allergy Testing Program on Antimicrobial Stewardship: A Multicenter Evaluation. Clin. Infect. Dis..

[42] Davenport L.A.P., Davey P.G., Ker J.S. (2005). An Outcome-Based Approach for Teaching Prudent Antimicrobial Prescribing to Undergraduate Medical Students: Report of a Working Party of the British Society for Antimicrobial Chemotherapy. J. Antimicrob. Chemother..

[43] Ha D.R., Haste N.M., Gluckstein D.P. (2019). The Role of Antibiotic Stewardship in Promoting Appropriate Antibiotic Use. Am. J. Lifestyle Med..

[44] Afzal Khan A.K., Gausia B., Reshma K.K. (2013). Antibiotic Resistance and Usage—A Survey on the Knowledge, Attitude, Perceptions and Practices among the Medical Students of a Southern Indian Teaching Hospital. J. Clin. Diagn. Res..

[45] Struzycka I., Mazinska B., Bachanek T., Boltacz-Rzepkowska E., Drozdzik A., Kaczmarek U., Kochanska B., Mielczarek A., Pytko-Polonczyk J., Surdacka A. (2019). Knowledge of Antibiotics and Antimicrobial Resistance amongst Final Year Dental Students of Polish Medical Schools—A Cross-sectional Study. Eur. J. Dent. Educ..

