# Supplementary material for: Self-Perception and Assessment of Antibiotic Therapy Knowledge in Dental Students in Spain: A Cross-Sectional Observational Study
Source: Antibiotics (Basel). 2025 Jul 27;14(8):755. doi: 10.3390/antibiotics14080755 (PMC12383009; doi:10.3390/antibiotics14080755)
Supplement: Supplementary file 1 [file antibiotics-14-00755-s001.zip › antibiotics-3706364-supplementary.pdf]

Questionnaire

1. **Gender**
   a) Male
   b) Female
2. **If you had completed your dental degree today, how confident would you feel in making an accurate diagnosis of an infection?** (1 = not at all confident, 5 = completely confident)
   a) 1
   b) 2
   c) 3
   d) 4
   e) 5
3. **If you had completed your dental degree today, how confident would you feel in prescribing antibiotics? (selecting the appropriate type, dosage, and duration)** (1 = not at all confident, 5 = completely confident)
   a) 1
   b) 2
   c) 3
   d) 4
   e) 5
4. **How confident would you feel managing a patient demanding antibiotic prescription when not clinically indicated?** (1 = not at all confident, 5 = completely confident)
   a) 1
   b) 2
   c) 3
   d) 4
   e) 5
5. **During your undergraduate dental training, do you think you received adequate/sufficient knowledge on antibiotic use in the following areas of dentistry?**

a) Endodontics<br>b) Oral Surgery<br>c) Periodontics<br>d) Oral Medicine<br>e) Paediatric Dentistry<br>f) Oral Implantology	Yes<br>No

6. **How many hours of antibiotic-related training do you estimate you received during your undergraduate dental education?**
   a) 0 – 10 h
   b) 11 – 20 h
   c) 21 – 40 h
   d) 41 – 60 h
   e) > 60 h

7. **What teaching methods were used for antibiotic education?**

a) Lectures<br>b) E-learning<br>c) Clinical scenarios/case-based learning<br>d) Direct patient care in clinical settings	Yes<br>No<br>No, but I believe it would be very useful

8. **To what extent did lectures influence your training and knowledge of antibiotics?** (1 = not at all, 5 = greatly)
   a) 1
   b) 2
   c) 3
   d) 4
   e) 5
9. **To what extent did clinical practice influence your training and knowledge of antibiotics?** (1 = not at all, 5 = greatly)
   a) 1
   b) 2
   c) 3
   d) 4
   e) 5
10. **To what extent has your personal interest in the topic of antibiotics—outside the academic setting—contributed to your knowledge and training in this area? (e.g., self-directed literature review, participation in webinars or related courses)** (1 = not at all, 5 = greatly)
    a) 1
    b) 2
    c) 3
    d) 4
    e) 5
11. **What percentage of antibiotic prescriptions in dentistry do you believe are unnecessary or could have been avoided?**
    a) < 1%
    b) 1 – 20%
    c) 21 – 40%
    d) 41 – 60%
    e) 61 – 80%
    f) 81 – 100%
12. **Do you think dentists play a key role in preventing antimicrobial resistance?** (1 = not at all, 5 = absolutely)
    a) 1
    b) 2
    c) 3
    d) 4
    e) 5

13. **Overall, do you believe you have been adequately trained on antibiotic use during your dental degree?**
    a) Yes
    b) No
14. **In an endodontic infection where systemic antibiotic prescription is indicated, which antibiotic would you prescribe in a patient with no known allergies?**
    a) Amoxicillin 500 mg
    b) Amoxicillin 750 mg
    c) Amoxicillin 1 g
    d) Amoxicillin/clavulanic acid 250/62.5 mg
    e) Amoxicillin/clavulanic acid 500/125 mg
    f) Amoxicillin/clavulanic acid 875/125 mg
    g) Clindamycin 300 mg
    h) Azithromycin 250 mg
15. **For how many days would you prescribe antibiotics for an endodontic infection?**
    a) < 3
    b) 3 – 5
    c) 6 – 7
    d) > 7
    e) Minimum of 3 days and until symptoms resolve.
16. **In an endodontic infection where systemic antibiotic prescription is indicated, which antibiotic would you prescribe for a patient allergic to penicillin?**
    a) Clindamycin 300 mg
    b) Azithromycin 250 mg
    c) Azithromycin 500 mg
    d) Azithromycin 1 g
    e) Metronidazole + spiramycin
    f) Erythromycin
    g) Lincomycin
    h) Other
17. **In which of the following clinical scenarios do you believe antibiotic prescription is indicated?**

a) Acute apical abscess with systemic symptoms (fever, malaise and/or lymphadenopathy)<br>b) Acute apical abscess progressing to cervicofacial cellulitis<br>c) Symptomatic irreversible pulpitis (pain but no other pulpal or periapical signs of infection)<br>d) Asymptomatic pulp necrosis (no pain on percussion or mastication)<br>e) Symptomatic apical periodontitis (spontaneous acute pain, percussion and mastication pain, widening of periodontal ligament space observed)<br>f) Asymptomatic apical periodontitis (periapical radiolucency, with or without sinus tract)<br>g) Acute apical abscess without systemic symptoms in healthy patients (localized swelling only)	Yes<br>No<br>Not sure

h)Acute apical abscess without systemic symptoms in immunocompromised patients (localized swelling only)<br>i) Reimplantation of an avulsed permanent tooth	

18. Were you aware that antiseptics can also lead to bacterial resistance?

a) Yes
b) No
